# Food Sources and Potential Determinants of Dietary Vitamin C Intake in Chinese Adults: A Cross-Sectional Study

**DOI:** 10.3390/nu10030320

**Published:** 2018-03-07

**Authors:** Xiaofang Jia, Zhihong Wang, Bing Zhang, Chang Su, Wenwen Du, Jiguo Zhang, Ji Zhang, Hongru Jiang, Feifei Huang, Yifei Ouyang, Yun Wang, Li Li, Huijun Wang

**Affiliations:** National Institute for Nutrition and Health, Chinese Center for Disease Control and Prevention, Beijing 100050, China; jiaxf@ninh.chinacdc.cn (X.J.); wangzh@ninh.chinacdc.cn (Z.W.); zhangbing@chinacdc.cn (B.Z.); suchang@ninh.chinacdc.cn (C.S.); duww@ninh.chinacdc.cn (W.D.); zhangjg@ninh.chinacdc.cn (J.Z.); zhangji@ninh.chinacdc.cn (J.Z.); jianghr@ninh.chinacdc.cn (H.J.); huangff@ninh.chinacdc.cn (F.H.); ouyyf@ninh.chinacdc.cn (Y.O.); wangyun@ninh.chinacdc.cn (Y.W.); lili@ninh.chinacdc.cn (L.L.)

**Keywords:** vitamin C, dietary intake, food sources, determinants, adults

## Abstract

Vitamin C is essential for human health. It is important to estimate the dietary vitamin C intake in the Chinese population to examine the effects of the nutritional transition occurred in recent decades. The present study aimed to estimate the dietary vitamin C intake in Chinese adults by using cross-sectional data from the 2015 China Nutritional Transition Cohort Study and selecting those aged 18–65 years with complete records of sociodemographic characteristics and dietary measurements (*n* = 11,357). Wilcoxon rank-sum test, Kruskal-Wallis analysis, Chi-squared test, and multiple logistic regression were employed to analyze the daily dietary vitamin C intake on the basis of three-day 24 h dietary recalls and food sources in relation to demographic factors, to evaluate vitamin C intake status using the estimated average requirement cut-off point, and to explore underlying influencing factors. The mean (SD (standard deviation)) and median (interquartile range) levels of the dietary vitamin C intake in adults were 78.1 (54.6) and 65.4 (61.4) mg/day, respectively. Light vegetables, dark vegetables, fruits, and tubers were the top four food sources, contributing a combined 97.3% of total daily dietary vitamin C intake in the study population. The prevalence of risk of insufficient dietary vitamin C intake was 65.1%. Both the distribution of vitamin C intake and the prevalence of risk of insufficient dietary vitamin C intake differed by several demographic factors. Educational level, residence area, geographic location, vegetable consumption, and total energy intake were independent determinants of the risk of insufficient dietary vitamin C intake. In conclusion, dietary vitamin C intake is inadequate in Chinese adult population, and an increase in vitamin C intake should be recommended especially to the population at risk for vitamin C insufficiency.

## 1. Introduction

Vitamin C, also known as ascorbic acid, is a water-soluble vitamin naturally present in some foods, particularly fruits and vegetables. It is used as a food additive and is available as a dietary supplement [1]. Because humans are unable to synthesize it, vitamin C is considered to be an essential dietary micronutrient [1]. The Chinese Dietary Reference Intakes recommend an estimated average requirement (EAR) for vitamin C for adults of 85 mg/day [2]. A regular and adequate intake is required to prevent vitamin C insufficiency.

Along with the rapid economic growth and urbanization observed in China over the past few decades, the Chinese population has experienced a nutritional transition characterized by dramatic changes in the dietary patterns, eating, and cooking behaviors [3,4]. Between 1991 and 2011, the consumption of animal source foods increased; over the same time frame, the consumption of coarse grains, legumes, and other healthy foods, e.g., vegetables, declined, and fruit intake remained low [5]. Consistent with these findings, the China Health and Nutrition Survey (CHNS, 1989–2009) showed a decrease in dark vegetable consumption in adults, accompanied by a significantly decreased dietary vitamin C intake, from 117.2 and 111.0 mg/day in 1989 to 85.2 and 83.1 mg/day in 2009 on average for males and females, respectively [6]. Results of the subsequent China National Nutrition and Health Survey (2010–2012) showed that the average dietary vitamin C intake in the Chinese population was 80.1 mg/day, and the mean probability of adequacy of vitamin C was 38.9%, suggesting that dietary vitamin C intake continued to be inadequate in most Chinese [7]. Compared with a reported range from 80 to 230 mg/day of mean vitamin C intake among 27 centers in 10 European countries [8], vitamin C intake in Chinese appeared low [8]. Thus, we must estimate vitamin C intake in the setting of the recent nutritional transition in China before we can recommend interventions to improve dietary vitamin C intake in the Chinese population.

Although fruits and vegetables remain the food groups with the highest contribution to total vitamin C intake [7,8,9], significant disparities in food availability, food choice, and consumption pattern among different ethnic groups worldwide could influence the primary food sources of vitamin C intake from one country to another [10]. In addition, the increasingly westernized lifestyle of China, characterized by the pronounced growth in the consumption of processed foods and beverages and a propensity to eat away from home [5], may lead to changes in the typical pattern of food sources of vitamin C in this country. Besides fruit and vegetable consumption, the role of sociodemographic factors, including income, educational level, and nutritional status [11], and the independent influence of smoking [12] in vitamin C status have been evaluated. In China, previous studies estimated the vitamin C intake stratified by gender, age, residence region, and household income level. However, independent roles of the sociodemographic factors and dietary habits have not been investigated yet, despite evidence that individuals from rural villages or households with low income and members of the aging population have a lower vitamin C intake [6,7,13].

Therefore, the present study aimed to estimate dietary vitamin C intake and vitamin C food sources and evaluate the prevalence of risk of inadequate vitamin C intake in the adult population using newly collected data from the China Nutritional Transition Cohort Study carried out in 2015. To identify independent determinants of the vitamin C intake status, this study further explored potential influencing factors. These findings would be valuable for developing and implementing public health strategies to meet the recommended vitamin C intake and provide guidance for population-specific nutrition education and intervention programs.

## 2. Materials and Methods

### 2.1. Study Population

Data in the present study were derived from the 2015 China Nutritional Transition Cohort Study conducted by National Institute for Nutrition and Health, Chinese Center for Disease Control and Prevention, which was an amplified survey based on the CHNS [4], an ongoing and longitudinal study established in 1989 by the Chinese Center for Disease Control and Prevention and the University of North Carolina at Chapel Hill. The covered provinces (municipalities) in each round of survey are shown in Figure S1. A multistage, random cluster process was used to draw a sample in each of the provinces (municipalities). The counties and cities in each province were stratified by income (low, middle, and high), and a weighted sampling scheme was used to randomly select four counties and two cities in each province (municipality). Villages and townships within the counties and urban and suburban neighborhoods within the cities were selected randomly. In each community, 20 households were randomly selected, and all household members were interviewed [4]. Compared to the previous surveys, an additional three provinces were included in this wave, according to the criteria of substantial variations in geography, economic development, public resources, and health indicators [4].

Subjects aged 18–65 years with complete data of sociodemographic characteristics, anthropometric measures, and dietary surveys were selected to participate in the present study. We excluded women who were pregnant (*n* = 56) and lactating (*n* = 108), and subjects with implausible energy intake (<500 or >5000 kcal/day, *n* = 63). For participants with dietary consumption values outside the sex-specific limits of dietary consumption distribution (<P_1_ or >P_99_, P: percentile) [1] including dietary vitamin C intake amount (*n* = 56 of <P_1_ and 56 of >P_99_ in males, *n* = 69 of <P_1_ and 69 of >P_99_ in females), fresh fruit consumption frequency (*n* = 49 of >P_99_ in males, *n* = 71 of <P_1_ and 65 of >P_99_ in females), and fresh vegetable consumption frequency (*n* = 50 of <P_1_ and 47 of >P_99_ in males, *n* = 62 of <P_1_ and 64 of >P_99_ in females), we used the corresponding sex-specific P_1_ and P_99_ to replace those of <P_1_ and >P_99_, respectively. A total of 11,357 participants were involved in the analysis (Figure S2). Written informed consent was obtained from all subjects before they participated in the study. The study was conducted in accordance with the Declaration of Helsinki, and the protocol was reviewed and approved by the Institutional Review Board of National Institute for Nutrition and Health (No. 2015017, 18 August 2015). 

### 2.2. Measurement of Dietary Intake

Detailed information, including amount consumed, cooking method, and eating location, on three meals and snacks for each individual, was obtained through a face-to-face interview of 24 h dietary recalls and recorded using a tablet device (Lenovo ThinkPad Tablet 2, Lenovo, Beijing, China) by trained staff across three consecutive days, including two weekdays and one weekend day [4,14,15].

### 2.3. Dietary Estimation and Food Sources of Vitamin C

Based on the intake amount of each ingredient of foods consumed during the survey period, the vitamin C content of the reported food items was coded according to the Chinese Food Composition Table [16]. Total vitamin C intake for each person was estimated by summing the vitamin C contribution of each food item and expressed as daily intake (mg/day).

The estimated vitamin C intake level was compared with that reported in the Chinese Dietary Reference Intakes [2], in which EAR and recommended nutrient intake (RNI) for vitamin C are 85 and 100 mg/day in adults aged 18–65 years, respectively. The proportion of subjects with a vitamin C intake <85 mg/day, indicating the prevalence of risk of insufficient dietary vitamin C intake in the study population, was calculated. The proportion of subjects with a vitamin C intake ≥100 mg/day, indicating those with less probability of insufficient dietary vitamin C intake, was evaluated.

Food items contributing to vitamin C intake were grouped according to the China Food Composition Table [16]. Food sources of vitamin C included five food groups, and the top four items were light vegetables (carotene amount ≤ 500 μg), dark vegetables (carotene amount > 500 μg), fruits, and tubers (potatoes), while the remaining thirty food groups were combined into one item because of minor contribution of each food group to vitamin C intake. The breakdown of fruit and vegetable contributors are listed in Table S1. The contribution percentage to total vitamin C intake from various food sources were calculated.

### 2.4. Dietary Estimation of Total Energy

Similarly to the dietary vitamin C estimation described in detail above, the energy content of each reported food item was coded according to the Chinese Food Composition Table on the basis of the individual dietary intake data during the survey period [16]. The total energy intake for each person was estimated by summing the energy contribution from each food item and was expressed as daily intake (kcal/day).

### 2.5. Assessment of Fresh Fruit and Vegetable Consumption Frequency

The participants were asked to complete a validated semi-quantitative food frequency questionnaire and report whether they consumed sixty-three foods and beverages during the last year and the frequency of that consumption, defined as the average number of times per day, week, month, or year, depending on whether the consumption was usual or unusual. The data on various fruit and vegetable consumption were used for the analysis. For each item, if the participant was a non-consumer, then his/her consumption frequency was set to zero time daily. For consumers, the reported average consumption frequency was converted into a uniform estimate of times/day by dividing times/week by 7, times/month by 30.5, and times/year by 365. Finally, the consumption frequency of each item was summed as that of fruits and vegetables, respectively, which was categorized into three levels (low: >0 and <0.3 times/day for fruits, >0 and <1.7 times/day for vegetables; moderate: ≥0.3 and <0.7 times/day for fruits, ≥1.7 and <2.9 times/day for vegetables; and high: ≥0.7 times/day for fruits, ≥2.9 times/day for vegetables) by tertiles.

### 2.6. Anthropometry

Height and body weight were measured by trained health workers on the basis of a standard protocol recommended by the World Health Organization [17]. The height and weight were collected using a portable stadiometer (SECA206, Seca, Hamburg, Germany) and the TANITA BC601 foot-to-foot body composition analyzer (Tanita Cooperation, Tokyo, Japan) when the participants were wearing light clothing and no shoes in the Community Health and Service Center. Height and weight were recorded to the nearest 0.1 cm or kg, respectively. The body mass index (BMI) was calculated by dividing the participants’ weight (kg) by their squared height (m) and the participants were divided into four categories according to the Working Group on Obesity in China recommended criteria, i.e., underweight: BMI < 18.5 kg/m^2^; normal: 18.5 ≤ BMI < 24 kg/m^2^; overweight: 24 ≤ BMI < 28 kg/m^2^; and general obesity: BMI ≥ 28 kg/m^2^ [18].

### 2.7. Measurement of Sociodemographic Characteristics

Questionnaires were employed to collect information for each subject in terms of age, gender, educational level, smoking and alcohol intake, household income, and residence region.

### 2.8. Statistical Analysis

Data analysis was performed by SAS 9.4 (SAS Inc., Cary, NC, USA). Continuous variables were presented as mean ± standard deviation (SD) and median (interquartile range), while categorical variables were expressed as *n* (%). Because of the non-normal distribution, the non-parametric Wilcoxon rank-sum test or the Kruskal-Wallis analysis were performed to test differences in the distribution of dietary vitamin C intake and the contribution percentage of food sources to the total vitamin C intake by subgroups of participants according to sociodemographic factors (age: 18–49 and 50–65 years; gender: male and female; educational level: primary school and below, middle school, high school and above; smoking status: never, former, and current; alcohol intake: no and yes; annual household income per capital: low (>0 and <2.4 thousand yuan/per capita), moderate (≥2.4 and <10.9 thousand yuan/per capita) and high (≥10.9 thousand yuan/per capita) by tertiles; residence area: city, suburban, town or county capital city, and rural village classed according to the administrative divisions; geographic location: north or south depending on the Qinling Mountains–Huihe River Line, an important geographic boundary; fruit and vegetable consumption frequency, and BMI status). If the difference was significant among more than two groups, an additional multiple comparison was conducted by the Student-Newman-Keuls (SNK) method. The prevalence of risk of insufficient dietary vitamin C intake in adults and the proportion of subjects with a lower risk for insufficient dietary vitamin C by sociodemographic factors were compared by the Chi-squared test. Further, multiple logistic regression and quantile regression were employed to explore the differential and independent effects of the sociodemographic variables on the prevalence of risk of insufficient dietary vitamin C intake in adults and on various areas of vitamin C intake distribution, respectively. The dependent variables were the dietary vitamin C intake category (<85 vs. ≥85 mg/day) and the dietary vitamin C intake level in the logistic regression model and in the quantile regression model, respectively. On the basis of the results of the aformentioned analysis of the effects of sociodemographic factors on the distribution of dietary vitamin C intake and on the proportion of subjects at risk for insufficient dietary vitamin C intake, and of the findings in previous reports [6,7,11,12,13], gender, educational level, residence area, geographic location, smoking status, alcohol intake, and vegetable consumption frequency were included as independent variables in the models. In addition, considering the underlying influence of dietary energy intake, the total daily energy intake (low: >0 and <1682 kcal/day, moderate: ≥1682 and <2247.54 kcal/day and high: ≥2247.54 kcal/day by tertiles) was added in the models. All independent variables were included simultaneously in both logistic and quantile regression models, and no collinearity was detected between variables (tolerance: 0.53–0.94). A value of *p* < 0.05 was considered significant. 

## 3. Results

### 3.1. Basic Characteristics of the Study Population

A sample of 11,357 adults aged 18–65 years was examined in the present study; those aged 18–49 years accounted for 55.4% (Table 1). About 56.5% of the subjects were females. The percentage of the population with a high school education or above was 37.8%. There were 74.7% and 71.6% of subjects without smoking and alcohol intake, respectively, similar to what reported elsewhere [14]. The frequency of the categories of high consumption of fruit and vegetables was 1.2 and 4.3 times/day, respectively. The prevalence of overweight and obesity in the study population was 34.7% and 13.8%, respectively. A larger proportion of subjects were from rural areas (46.1%) and the southern part of China (62.3%).

### 3.2. Dietary Vitamin C Intake Level by Sociodemographic Factors

The mean and median levels of dietary vitamin C intake in all subjects were 78.1 and 65.4 mg/day, respectively (Table 1). Gender, educational level, residence area, geographic location, smoking status, alcohol intake, and vegetable consumption frequency significantly influenced the distribution of the daily dietary vitamin C intake (all *p* < 0.05). The daily vitamin C intake was higher in males, in subjects from the southern part of China, and in those who drank alcohol than in their respective counterparts. Subjects with education at the middle school level (mean: 80.4 mg/day; median: 67.1 mg/day) had larger vitamin C intake than those with primary school education or below (mean: 75.5 mg/day; median: 63.9 mg/day, *p* < 0.05), but no difference in vitamin C intake was found compared with those having a high school education or above (mean: 77.8 mg/day; median: 65.3 mg/day). The daily vitamin C intake was higher in subjects from suburban areas than in those from any other residence areas. The daily dietary vitamin C intake was significantly different and presented an increasing trend associated with increased vegetable consumption frequency.

### 3.3. Prevalence of Risk of Insufficient Dietary Vitamin C Intake in the Study Population and Proportion of Subjects with a Lower Risk of Insufficient Dietary Vitamin C in Relation to Sociodemographic Factors

Overall, the prevalence of risk of insufficient dietary vitamin C in the study population was 65.1%, and the proportion of subjects with a lower risk of insufficient dietary vitamin C intake was 25.9% (Table 2), both of which significantly differed by gender, educational level, residence area, geographic location, smoking status, alcohol intake, and vegetable consumption (all *p* < 0.05). The prevalence of risk of inadequate vitamin C was greater, and the proportion of subjects with a lower likelihood of insufficient vitamin C was lower in females, in subjects living in northern China, and in those without alcohol intake, than in their respective counterparts. Among subgroups, the proportion of subjects at risk of insufficient dietary vitamin C seemed to be lower, and the proportion of subjects with less probability of insufficient dietary vitamin C intake was likely to be higher, in participants with middle school education, in those from suburban areas, in smokers, and in those with great vegetable consumption frequency. Moreover, the average levels of daily energy, protein, fat, and carbohydrate intake were 1940.9 kcal/day, 62.0 g/day, 77.0 g/day, and 245.4 g/day, respectively, in subjects with <85 mg/day of vitamin C intake, while those in subjects with ≥85 mg/day of vitamin C intake were 2273.3 kcal/day, 75.2 g/day, 92.4 g/day, and 279.5 g/day, respectively.

### 3.4. Contribution Percentages of Food Sources to the Total Dietary Vitamin C Intake in Relation to Sociodemographic Factors

**Figure 1:**
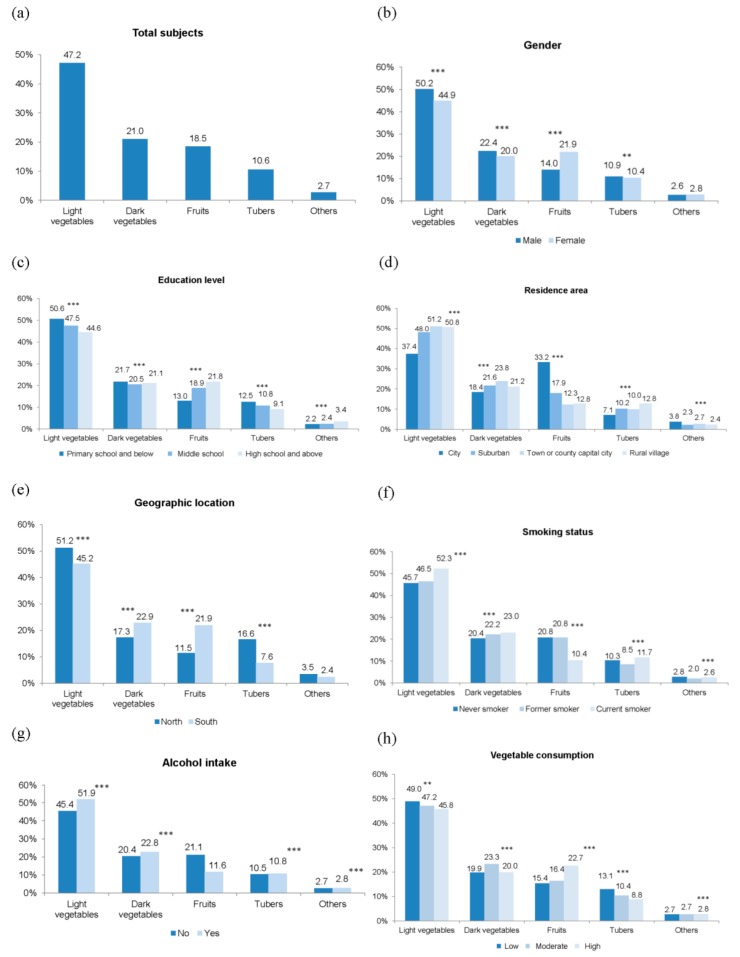
Contribution percentage of food sources to the total vitamin C intake by sociodemographic factors. (**a**) Total subjects; (**b**) subgroups by gender; (**c**) educational level; (**d**) residence area; (**e**) geographic location; (**f**) smoking status; (**g**) alcohol intake; (**h**) vegetable consumption. ** *p* < 0.01, and *** *p* < 0.001 indicate significant differences in the distribution of the contribution percentages of food sources to the total vitamin C intake by sociodemographic factors by Wilcoxon rank-sum test or Kruskal-Wallis analysis.

The top four food sources were light vegetables, dark vegetables, fruits, and tubers in Chinese adults, and the contribution to the total daily dietary vitamin C intake was 47.2%, 21.0%, 18.5%, and 10.6%, respectively (Figure 1a). The patterns of contributions of the various food sources in the subgroups according to sociodemographic factors were highly similar to those of the total study population, and significant differences in the distribution of their contribution percentages were observed (Figure 1b–h). The contribution percentages of light and dark vegetables to the total vitamin C intake in males (50.2% and 22.4%) were higher than those in females (44.9% and 20.0%), while that of fruits in females was 1.6 times greater than in males (all *p* < 0.001, Figure 1b). Light vegetables, dark vegetables, and tubers individually contributed a higher proportion to the total vitamin C intake in adults with education of primary school and below, relative to those with education of middle school or high school and above, totally accounting for 84.8%. However, the contribution of fruits was greater in adults with high school education and above (21.8%), and lower in those with primary school and below (13.0%, Figure 1c). There were considerable differences in the contributions of the different food sources to total vitamin C among adults from various residence areas, especially for light vegetables and fruits (Figure 1d). Light vegetables and tubers contributed largely to vitamin C intake in participants from the northern part of China, while dark vegetables and fruits accounted for higher proportions in southern participants (Figure 1e). Current smokers had a greater vitamin C intake from light and dark vegetables and a lower intake from fruits compared to never and former smokers (Figure 1f). Alcohol consumers had a higher vegetables contribution percentage to vitamin C intake (*p* < 0.001), but a partially lower fruit contribution percentage (*p* = 0.057) than their counterparts (Figure 1g). Light vegetables and tubers were more important vitamin C contributors (49.0% and 13.1%) in adults with a low vegetable consumption frequency than in those with moderate (47.2% and 10.4%) and high (45.8% and 8.8%) consumption frequencies. The percentage of contribution from fruits (22.7%) was the highest in the group with high vegetable consumption frequency (Figure 1h). 

There were significant effects of age, household income, fruit consumption, and BMI status on the distribution of the contribution percentages of various food sources to the total vitamin C intake (Figure S3a–d). The contribution of vegetables was higher in the young group than in the old group, but that of fruits was larger in the latter. Fruit contribution to the total vitamin C intake was largest in adults from households with high income. Both light and dark vegetables contributed higher percentages to vitamin C intake in adults with low fruit consumption than in either of their counterparts, while the findings from fruits were the opposite. The contribution of the various food sources to vitamin C intake was different in relation to the BMI status.

### 3.5. Potential Determinants of the Dietary Vitamin C Intake Status

To explore independent influencing factors of the dietary vitamin C intake status, we performed multiple logistic regression by including gender, education level, residence area, geographic location, smoking status, alcohol intake, vegetable consumption, and total daily energy intake as covariates (Table 3). After adjustment, in comparison with subjects living in cities, those living in towns or county capital cities (odds ratio (OR): 1.20, 95% Confidence Interval (CI): 1.05–1.37) and in rural villages (OR: 1.17, 95% CI: 1.04–1.32) had a significantly higher risk of insufficient dietary vitamin C intake. Conversely, educational level of middle school (OR: 0.84, 95% CI: 0.76–0.93), suburban areas of residence (OR: 0.72, 95% CI: 0.63–0.82), southern part of China (OR: 0.85, 95% CI: 0.78–0.92), high vegetable consumption frequency (OR: 0.68, 95% CI: 0.62–0.75), and moderate (OR: 0.60, 95% CI: 0.54–0.66) and high (OR: 0.33, 95% CI: 0.30–0.37) total daily energy intake were protective factors for the risk of insufficient dietary vitamin C intake.

The quantile regression model, using dietary vitamin C intake as the dependent variable and gender, educational level, residence area, geographic location, smoking status, alcohol intake, vegetable consumption, and total daily energy intake as independent variables, was applied to investigate the influences of various sociodemographic factors on the distribution of dietary vitamin C intake in the study population. The selected results of 10th, 25th, 50th, 75th, and 90th percentiles are presented in Table S2. Consistent with the findings of the multiple logistic regression, after adjusting for covariates, the influence of particular variables, including gender, educational level, residence area, geographic location, vegetable consumption frequency, and total daily energy intake, was found to vary significantly across the intake distribution. 

## 4. Discussion

On the basis of the antioxidant property of vitamin C, increasing evidence indicates that vitamin C intake is essential for human health by reducing the risk of progression to cardiovascular disease, type 2 diabetes mellitus, or other diseases in which oxidative stress plays a pivotal role [1,19]. Understanding dietary vitamin C intake and the extent to which the intake does not meet the recommendations and the underlying influencing factors will be valuable for targeted interventions to assure adequate vitamin C intake. The present study, which used three-day 24 h dietary recalls, found that the average vitamin C intake was 78.1 mg/day in adults aged 18–65 years, and the prevalence of risk of insufficient dietary vitamin C intake in the study population was 65.1%. The top four food sources of vitamin C were light and dark vegetables, fruits, and tubers, contributing a combined 97.3% of the total vitamin C. After fully adjusting for covariates, the subjects living in towns or rural villages resulted in potentially vulnerability to the risk of insufficient vitamin C intake, while the subjects having middle school education, those living in suburban regions or southern regions, those with a high vegetable consumption frequency, and those with a moderate and high total daily energy intake were protected against the risk of insufficient dietary vitamin C intake. The independent influences of educational level, residence area, geographic location, vegetable consumption frequency, and total daily energy intake were relatively strong in the areas of lower intake of vitamin C intake distribution, which is of great concern. Like all dietary methods, 24 h recalls are subject to random errors that lower the precision and to systematic errors that can reduce the accuracy at each stage of the measurement protocol [20]. Here, although our study was well designed to reduce random errors and systematic errors by incorporating standardized quality control procedures and collecting 24 h recalls per person across three consecutive days, including two weekdays and one weekend day, dietary vitamin C underreporting and some degree of misclassification might have been possible, which should be taken into account when considering the findings in the present study.

China National Nutrition and Health Survey in 1982, 1992, 2002, and 2010–2012 reported that the average dietary vitamin C intake in the Chinese population was 129.4, 100.2, 88.4, and 80.1 mg/day, suggesting a progressive decline in vitamin C intake over the past three decades [7,13]. The mean vitamin C intake (78.1 mg/day) in adults in 2015 in the present study seemed to be slightly lower than the above-mentioned findings from 2010–2012 nationwide. To evaluate the changes in vitamin C intake among the same population between different survey periods, we further focused on vitamin C intake in adults aged 18–49 years from nine provinces in the present study, which were 87.4 mg/day in males and 81.0 mg/day in females on average, and thus considerably lower than that in 1989 (117.2 mg/day for males and 111.0 mg/day for females), and showing a slightly increasing trend in males and a decreasing trend in females relative to vitamin C intake in 2009 (85.2 mg/day for males and 83.1 mg/day for females) [6]. Thus, it is evident that the dietary vitamin C intake in Chinese adults over the periods studied significantly decreased at the same time of the nutritional transition in China, and the intake in recent years has remained low and needs to be improved. Vitamin C intake from foods varied from about 80 to over 230 mg/day in adults in 10 European countries in a survey during 1995–2000 [8]; that of the US adult population in 2001–2002 ranged from 101.8 to 116.2 mg/day [21], and the most recent estimate for the Spanish population in 2013 was 84.4 mg/day [9]. The intake amount in the Chinese population appears to be lower than that of western countries. These differences may result from ethnic disparities in dietary patterns and nutrient intake from meals, largely related to portion size, recipe, and cooking method [22].

Around 38.9% of Chinese population had adequate vitamin C intake in the China National Nutrition and Health Survey 2010–2012, indicating more than 60% of the population was at risk of vitamin C inadequacy [7]. The present study showed that the prevalence of risk of insufficient dietary vitamin C intake in adults was 65.1% by using EAR cut-off point, partially consistent with the aforementioned estimation of the previous study [7]. In our study, the proportion of subjects with a lower likelihood of inadequate vitamin C intake using RNI was 25.9%. The specific recommendations for vitamin C intake vary in different countries. The reported percentage of the population not meeting the recommended vitamin C intake was 36% in the Spanish population [9], and 31% in the US population [21], which is significantly lower than what we found in our study, and likely due in part to disparities in fruit and vegetable consumption. There were increased trends in daily fruit and vegetable consumption in European and North American countries from 2002 to 2010 [23]; conversely, an unbalanced diet was adopted by the Chinese population, especially by middle-aged residents, characterized as high in energy-dense, nutrient-poor foods and low in fruit and vegetables [24,25]. Here, based on comparisons of dietary intake to EAR and RNI for vitamin C, we found that the dietary vitamin C intake in Chinese adults is a potential problem and needs to be addressed.

Indeed, fruits and vegetables are by far main contributors to dietary vitamin C. These two groups provided about 87% to the total vitamin C intake in the total population assessed in the present study, an estimate that tends to be higher than that reported in Spanish (70%) [9], Polish (75%) [26], and other European populations (80%) [8]. Additionally, the most important source of vitamin C was fruits in the US [10]. Conversely, vegetables ranked first in Chinese (68.2%) and European (19.9–42.9%) populations, and in China it was characterized by a higher contribution of light greens (47.2%) as the consumption of dark greens gradually decreased [6]. Fruit contribution in China (18.5%) was less than in the US (around 50.0%) and in European countries (23.3–59.3%) [8,10]. Therefore, we found that vegetables are the most important food source of vitamin C and fruit consumption is lower than vegetable consumption in the Chinese population. Consistent with other countries [8,26], tubers were also an important contributor to total vitamin C and ranked fourth in the Chinese adult population in the present study. Previous studies have found beverages ranked third or fourth among contributors of total vitamin C intake in subjects from the US [10] and European countries [8,9,26]; however, in the present study, drinks afforded a negligible proportion (data not shown), and the largest contribution observed was only 1.2% in subjects living in northern parts of China. Taken together, different patterns of food source contributions to total vitamin C were observed in China and western countries. Interestingly, the present study also observed a differentiated percent contribution of food sources in relation to sociodemographic factors, regarding especially fruit contributions.

The 15 provinces surveyed in the present study (Figure S1) vary substantially in geography, economic development, public resources, and health indicators. The nutrition and health behaviors and outcomes of each participant are influenced by changes in community organizations and programs and by changes in types of household and individual economic, demographic, and social factors. All these factors largely contributes to the skewed distribution of dietary vitamin C in the study population. Previous studies in China found that vitamin C intake was lower in populations from rural villages or households with lower income, as well as in the aging population [6,7,13]. This is the first study exploring the potential influencing factors behind these observations. Both the distribution of vitamin C intake and the prevalence of risk of insufficient dietary vitamin C intake in adults significantly differed by gender, educational level, residence region, geographic location of residence, smoking, alcohol intake, and vegetable consumption frequency. Educational level, residence region, geographic location, vegetable consumption, and total energy daily intake were further identified as independent indicators for the risk of vitamin C inadequacy in Chinese adults. The availability of fruit and vegetables was linked to favorable vitamin C intake in adults [27]. We investigated the impacts of fruit and vegetable consumption on vitamin C intake and found that a high frequency of vegetable consumption independently protected against the risk of dietary vitamin C inadequacy. No significance was observed for fruit consumption, which may be due to the lack of fruit consumption on a daily basis among more than 66% of the population on the basis of the findings of fruit consumption frequency in the present study. Similar to the findings in European countries [8], a clear geographic gradient of vitamin C intake was observed in our study, with higher intakes and negative association with the risk of vitamin C inadequacy in the southern regions as compared to the northern regions. In contrast to residency in suburban areas, residency in both town and rural villages increased the risk of insufficient dietary vitamin C. All these findings indicate that the vitamin C intake status may depend on parameters reflecting economic level, dietary habit, and food availability in the population from various regions. The association between gender and vitamin C intake varied [8,26]. Here, gender was not found to associate with the risk of insufficient dietary vitamin C intake after considering total energy intake in the model. Some evidence indicated the inverse association between smoking and vitamin C intake, and smoking was negatively associated with the preference for foods rich in vitamin C [8,12]. Conversely, only fruit contribution in current smokers in the present study was lower than in either never or former smokers, according to percent contributions of food sources to vitamin C intake, but this was not sufficient to match the higher vitamin C intake from vegetables and tubers. The vitamin C intake distribution significantly differed depending on the smoking status, in that smokers tended to have higher vitamin C intake; however, an independent influence of smoking on vitamin C was not observed in our study. The association between vitamin C intake and alcohol intake was similar to that observed for smoking. It was suspected that the different vitamin C intake distributions associated with smoking and alcohol intake in the present study were linked to a significant higher proportion of males in the smoker and drinker groups relative to their counterparts, and to the observed vitamin C intake variation in relation to gender found in our study. Regarding the impacts of smoking and alcohol intake on vitamin C-enriched foods and vitamin C intakes, future study is still required.

This is a representative and large study evaluating the intake and food sources of vitamin C across sociodemographic factors and exploring independent determinants for vitamin C intake, using data of adults from 15 provinces in China. However, the findings cannot be extrapolated to the general population. Another limitation of this study is that the 24 h dietary recalls for three consecutive days were generally collected in summer, and seasonal variations in food consumption rich in vitamin C could not be taken into account. Moreover, there are always recall biases and a potential underestimation in dietary recalls being self-reported measurements. In addition, this study considers only intakes of vitamin C from dietary sources. Dietary supplements are likely to be considerable sources of vitamin C in some particular populations. It should be borne in mind that the users of supplements may differ from non-users with regard to demographic characteristics [28].

## 5. Conclusions

Vitamin C intake in Chinese adults deviates from the recommended level, resulting in a high prevalence of risk of insufficient dietary vitamin C intake, which exerts potentially adverse influences on human health. Considering that fruit and vegetables are the main sources of vitamin C, the Chinese population should be encouraged to consume these foods according to dietary guidelines [29], especially those at high risk of insufficient dietary vitamin C intake.

## Figures and Tables

**Table 1:** Sociodemographic characteristics and daily dietary vitamin C intake in adults.

Parameters	*N* (%)	Vitamin C Intake (mg/Day)		*p-*Value
		Mean ± SD	Median (Interquartile Range)	
Total subjects	11,357 (100)	78.1 ± 54.6	65.4 (61.4)	
Age group (years)				0.069
18–49	6290 (55.4)	76.9 ± 53.1	64.7 (60.6)	
50–65	5067 (44.6)	79.5 ± 56.4	66.3 (62.2)	
Gender				0.001
male	4944 (43.5)	79.4 ± 53.7	67.6 (64.1)	
female	6413 (56.5)	77.1 ± 55.3	63.9 (59.3)	
Education level				0.003
primary school and below ^a^	3070 (27.0)	75.5 ± 52.6	63.9 (58.1)	
middle school ^b,c^	3998 (35.2)	80.4 ± 56.2	67.1 (66.1)	
high school and above ^a,c^	4289 (37.8)	77.8 ± 54.8	65.3 (59.9)	
Annual household income level (thousand yuan/per capital) ^1^				0.463
low	0.9 ± 0.7	77.6 ± 54.8	64.7 (63.4)	
moderate	6.1 ± 2.4	78.4 ± 54.0	66.1 (60.7)	
high	27.6 ± 19.1	78.3 ± 55.1	65.3 (59.8)	
Residence area				<0.001
city ^a^	2276 (20.0)	81.2 ± 61.7	65.8 (65.4)	
suburban ^b^	1865 (16.4)	87.9 ± 57.5	75.2 (69.8)	
town or county capital city ^c^	1979 (17.4)	72.7 ± 49.3	60.5 (57.7)	
rural village ^a,c^	5237 (46.1)	75.3 ± 51.6	64.2 (57.6)	
Geographic location				<0.001
north	4283 (37.7)	73.8 ± 51.7	62.2 (56.7)	
south	7074 (62.3)	80.7 ± 56.2	67.5 (64.6)	
Smoking status				0.015
never smoker	8482 (74.7)	77.6 ± 55.1	64.6 (60.0)	
former smoker	225 (2.0)	81.9 ± 53.4	68.7 (62.1)	
current smoker	2650 (23.3)	79.5 ± 53.1	68.1 (66.2)	
Alcohol intake				<0.001
no	8130 (71.6)	77.3 ± 55.2	64.2 (60.3)	
yes	3227 (28.4)	80.1 ± 53.1	68.8 (64.5)	
Fruit consumption (times/day) ^1^				0.073
low	0.2 ± 0.1	78.3 ± 52.7	66.0 (61.9)	
moderate	0.5 ± 0.1	76.9 ± 54.7	64.5 (60.4)	
high	1.2 ± 0.4	79.1 ± 56.4	66.2 (61.6)	
Vegetable consumption (times/day) ^1^				<0.001
low ^a^	1.2 ± 04	71.4 ± 50.2	60.5 (55.9)	
moderate ^b^	2.3 ± 0.3	77.9 ± 53.6	65.4 (59.7)	
high ^c^	4.3 ± 1.3	85.0 ± 58.8	71.6 (68.6)	
Body mass index (kg/m^2^)				0.084
<18.5	469 (4.1)	76.2 ± 52.8	65.9 (58.2)	
18.5–24	5388 (47.4)	76.8 ± 52.7	64.6 (61.6)	
24–28	3938 (34.7)	79.1 ± 57.0	65.3 (62.1)	
≥28	1562 (13.8)	80.8 ± 55.3	68.3 (59.5)	

^1^ Data are expressed as mean ± SD (standard deviation); Wilcoxon rank-sum test or Kruskal-Wallis analysis was performed to test the difference of the distribution of dietary vitamin C by sociodemographic factors; subgroups with different superscript letters were significantly different by multiple comparison of SNK (Student-Newman-Keuls method).

**Table 2:** Proportion of subjects at risk of insufficient dietary vitamin C intake and proportion of subjects with a lower risk of insufficient dietary vitamin C by sociodemographic factors.

Parameters	Subjects at Risk of Insufficient Dietary Vitamin C Intake (<85 mg/Day)		Subjects with a Lower Likelihood of Inadequate Dietary Vitamin C Intake (≥100 mg/Day)	
	*N* (%)	*p*-Value	*N* (%)	*p*-Value
Total subjects	7396 (65.1)		2938 (25.9)	
Age group (years)		0.367		0.193
18–49	4119 (65.5)		1597 (25.4)	
50–65	3277 (64.7)		1341 (26.5)	
Gender		<0.001		0.001
male	3118 (63.1)		1360 (27.5)	
female	4278 (66.7)		1578 (24.6)	
Education level		<0.001		<0.001
primary school and below	2071 (67.5)		733 (23.9)	
middle school	2512 (62.8)		1123 (28.1)	
high school and above	2813 (65.6)		1082 (25.2)	
Household income level		0.612		0.220
low	2460 (65.0)		1003 (26.5)	
moderate	2453 (64.8)		978 (25.8)	
high	2483 (65.6)		957 (25.3)	
Residence area		<0.001		<0.001
city	1460 (64.2)		615 (27.0)	
suburban	1059 (56.8)		633 (33.9)	
town or county capital city	1359 (68.7)		452 (22.8)	
rural village	3518 (67.2)		1238 (23.6)	
Geographic location		<0.001		<0.001
north	2948 (68.8)		953 (22.3)	
south	4448 (62.9)		1985 (28.1)	
Smoking status		0.003		0.002
never smoker	5599 (66.0)		2124 (25.0)	
former smoker	137 (60.9)		67 (29.8)	
current smoker	1660 (62.6)		747 (28.2)	
Alcohol intake		<0.001		0.002
no	5375 (66.1)		2039 (25.1)	
yes	2021 (62.6)		899 (27.9)	
Fruit consumption		0.648		0.749
low	2432 (64.7)		1002 (26.6)	
moderate	2558 (66.5)		949 (24.7)	
high	2406 (64.2)		987 (26.3)	
Vegetable consumption		<0.001		<0.001
low	2659 (70.3)		798 (21.1)	
moderate	2506 (66.2)		958 (25.3)	
high	2231 (58.9)		1182 (31.2)	
Body mass index (kg/m^2^)		0.208		0.536
<18.5	314 (67.0)		117 (25.0)	
18.5–24	3522 (65.4)		1386 (25.7)	
24–28	2562 (65.1)		1025 (26.0)	
≥28	998 (63.9)		410 (26.3)	

Chi-squared test was performed to generate the *p*-values.

**Table 3:** Association of the risk of insufficient dietary vitamin C intake (<85 mg/day) with sociodemographic factors using the multiple logistic regression model.

Independent Variables	<85 vs. ≥85 mg/Day of Dietary Vitamin C Intake	
	OR (95% CI)	*p*-Value
Gender		
male	1.0	
female	0.93 (0.83, 1.04)	0.205
Education level		
primary school and below	1.0	
middle school	0.84 (0.76, 0.93)	0.001
high school and above	0.95 (0.85, 1.07)	0.406
Residence area		
city	1.0	
suburban	0.72 (0.63, 0.82)	<0.001
town or county capital city	1.20 (1.05, 1.37)	0.007
rural village	1.17 (1.04, 1.32)	0.009
Geographic location		
north	1.0	
south	0.85 (0.78, 0.92)	<0.001
Smoking status		
never smoker	1.0	
former smoker	0.90 (0.67, 1.20)	0.470
current smoker	0.97 (0.86, 1.09)	0.582
Alcohol intake		
no	1.0	
yes	1.00 (0.89, 1.11)	0.924
Vegetable consumption		
low	1.0	
moderate	0.91 (0.82, 1.01)	0.064
high	0.68 (0.62, 0.75)	<0.001
Total daily energy intake		
low	1.0	
moderate	0.60 (0.54, 0.66)	<0.001
high	0.33 (0.30, 0.37)	<0.001

The logistic regression model was employed, considering gender, educational level, residence area, geographic location, smoking status, alcohol intake, vegetable consumption, and total daily energy intake as independent variables. OR: odds ratio; CI: confidence interval.

## Supplementary Materials

The following are available online at http://www.mdpi.com/2072-6643/10/3/320/s1, Figure S1: The geographic map of China to show the covered provinces (municipalities) in the China Nutritional Transition Cohort Study; Figure S2: Flow chart of the participants for the estimation of dietary vitamin C intake and its food sources and potential determinants in Chinese adults using cross-sectional data from 2015 China Nutritional Transition Cohort Study; Figure S3: Contribution percentage of food sources to the total vitamin C intake by sociodemographic factors; Table S1: The breakdown of fruit and vegetables contributing to dietary vitamin C intake; Table S2: Association of dietary vitamin C intake with sociodemographic factors using the quantile regression model.
