# Supplementary material for: Food Sources and Potential Determinants of Dietary Vitamin C Intake in Chinese Adults: A Cross-Sectional Study
Source: Nutrients. 2018 Mar 7;10(3):320. doi: 10.3390/nu10030320 (PMC5872738; doi:10.3390/nu10030320)
Supplement: Supplementary file 1 [file nutrients-10-00320-s001.pdf]

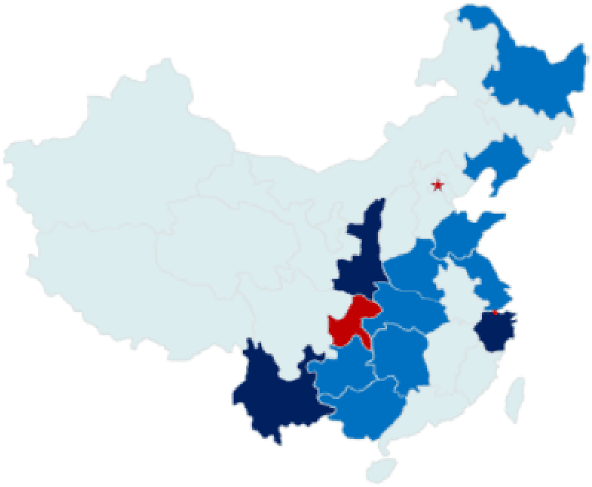

Figure S1. The geographic map of China to show the covered provinces (municipalities) in the China Nutritional Transition Cohort Study. The China Nutritional Transition Cohort Study conducted in 2015 is based on the China Health and Nutrition Survey, an ongoing and longitudinal study across nine diverse provinces (Heilongjiang, Liaoning, Shandong, Henan, Jiangsu, Hubei, Hunan, Guizhou, and Guangxi in light blue) and eight rounds of survey between 1989 and 2009. In 2011, the three largest municipal cities (Beijing in red pentagram, Shanghai in red spot, and Chongqing in red area) were added. An additional three provinces (Zhejiang, Shanxi, and Yunnan in dark blue) were included in 2015.

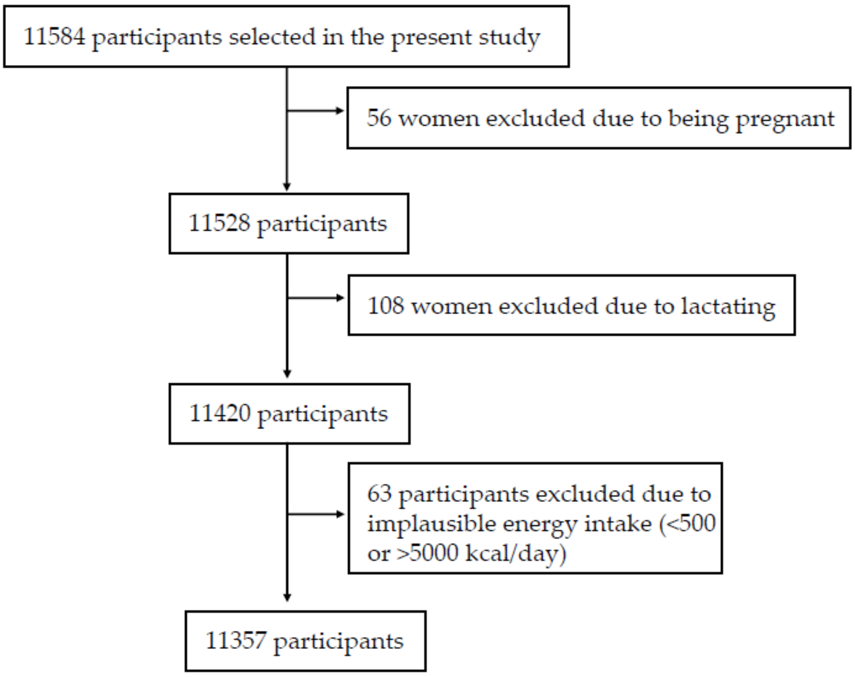

Figure S2. Flow chart of participants for the estimation of dietary vitamin C intake, and its food sources and potential determinants in Chinese adults using cross-sectional data from 2015 China Nutritional Transition Cohort Study.

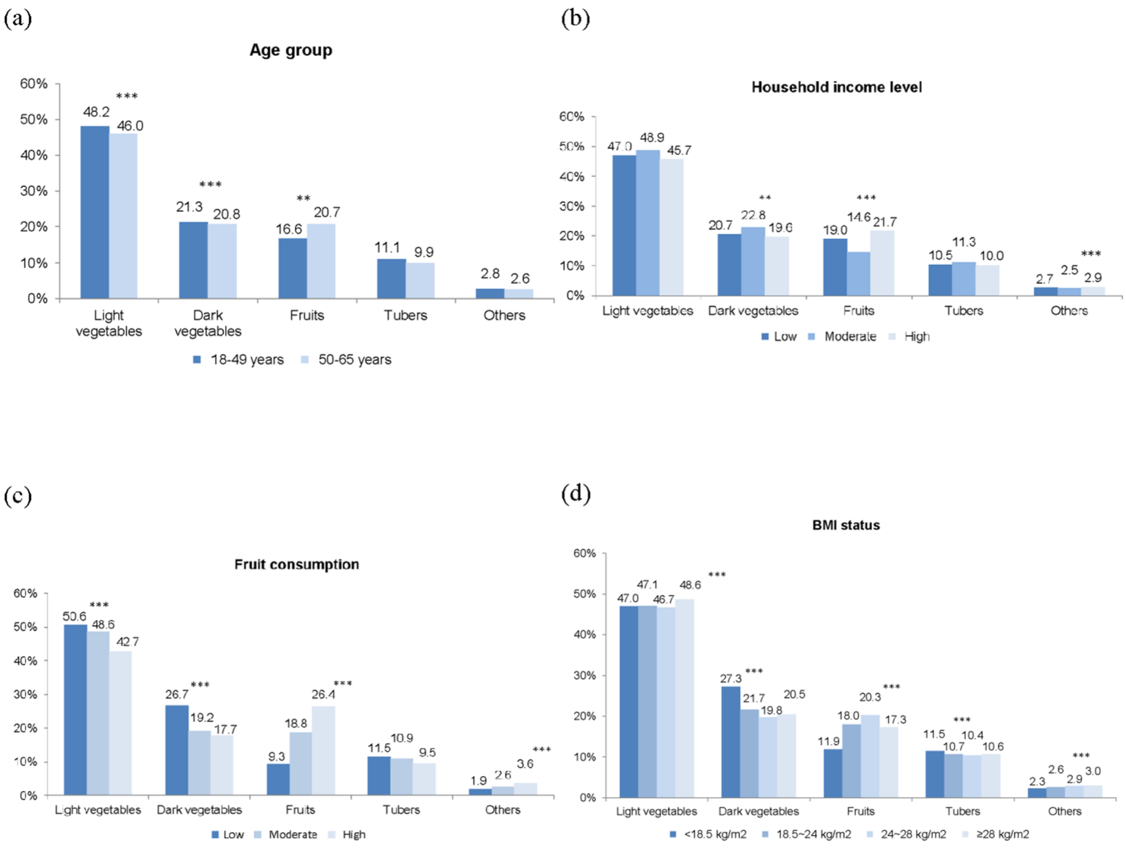

Figure S3. Contribution percentage of food sources to total vitamin C intake by socio-demographic factors. Subgroups by age (a), household income level (b), fruit consumption (c), and BMI status (d). **p<0.01, and ***p<0.001 indicate significant differences in the distribution of contribution percentage of food sources to total vitamin C intake by socio-demographic factors by Wilcoxon rank-sum test or Kruskal-Wallis analysis. BMI, body mass index.

Table S1. The breakdown of fruit and vegetables contributing to dietary vitamin C intake.

Group	Sub-group	Examples of food
Fruit and fruit products	Kernel fruit	apple, pear
	Drupe fruit	date, peach
	Berry	grape, pomegranate, persimmon
	Orange fruit	orange, pomelo
	Tropic fruit	banana, mango
	Melons	watermelon, casaba
Vegetables and vegetable products	Root vegetable	carrot, daikon, turnip
	Leguminous vegetable and sprout	kidney bean, yardlong, pea greens
	Cucurbitaceous and solanaceous vegetable	cucumber, zucchini, eggplant
	Allium vegetable	garlic stalk, scallion, garlic bulb
	Stem, leafy, and flowering vegetable	bok choi, cauliflower, cabbage
	Aquatic vegetable	lotus root, wildrice stem, water chestnut
	Tuber	taro, ginger, yam
	Wild vegetable	common spiderflower, balloonflower

Table S2 Association of dietary vitamin C intake with socio-demographic factors using quantile regression model.

**Independent variables**	**Quantile regression estimates**				
	**P_{10} (23.6 mg/day)**	**P_{25} (40.3 mg/day)**	**P_{50} (65.4 mg/day)**	**P_{75} (101.7 mg/day)**	**P_{90} (145.9 mg/day)**
Gender					
male	-0.89 (-2.74, 0.96)	**-2.96 (-5.10, -0.83)**	**-4.46 (-7.12, -1.79)**	-3.46 (-7.24, 0.32)	-3.17 (-10.18, 3.85)
Education level					
primary school and below	**-2.41 (-4.34, -0.48)**	**-3.15 (-5.28, -1.02)**	-0.99 (-3.78, 1.79)	2.22 (-1.26, 5.69)	2.57 (-4.45, 9.60)
middle school	-1.33 (-2.94, 0.27)	-1.59 (-3.64, 0.46)	2.32 (-0.50, 5.13)	**7.41 (4.00, 10.83)**	**6.35 (0.20, 12.49)**
Residence area					
city	0.17 (-2.14, 2.47)	-0.61 (-3.07, 1.86)	0.54 (-2.31, 3.40)	**5.41 (1.39, 9.44)**	**13.87 (5.93, 21.80)**
suburban	**5.09 (2.88, 7.29)**	**7.96 (5.31, 10.61)**	**10.98 (7.73, 14.24)**	**17.86 (13.89, 21.84)**	**18.79 (11.94, 25.64)**
town or county capital city	-0.69 (-2.51, 1.12)	-0.19 (-1.84, 1.47)	**-3.55 (-6.40, -0.70)**	-0.98 (-4.47, 2.51)	0.25 (-6.50, 7.01)
Geographic location					
north	-0.66 (-2.00, 0.68)	**-2.53 (-4.20, -0.85)**	**-2.71 (-4.83, -0.59)**	**-3.48 (-6.30, -0.66)**	-4.22 (-9.66, 1.22)
Smoking status					
never smoker	1.01 (-1.24, 3.26)	0.22 (-2.30, 2.73)	0.48 (-2.77, 3.74)	-4.13 (-8.34, 0.09)	1.07 (-6.50, 8.64)
former smoker	1.13 (-5.58, 7.83)	2.64 (-3.12, 8.39)	5.92 (-2.65, 14.49)	2.45 (-7.25, 12.15)	-4.35 (-24.59, 15.90)
Alcohol intake					
no	0.28 (-1.50, 2.06)	-1.44 (-3.63, 0.76)	**-3.07 (-5.86, -0.28)**	1.03 (-2.53, 4.58)	1.16 (-5.32, 7.63)
Vegetable consumption					
low	**-4.49 (-6.07, -2.91)**	**-4.22 (-6.01, -2.43)**	**-8.71 (-11.42, -6.01)**	**-12.18 (-15.48, -8.89)**	**-18.98 (-25.11, -12.84)**
moderate	-1.58 (-3.34, 0.17)	-1.47 (-3.41, 0.47)	**-7.13 (-9.90, -4.36)**	**-7.07 (-10.82, -3.32)**	**-7.78 (-14.28, -1.28)**
Total daily energy intake					
low	**-9.81 (-11.37, -8.25)**	**-15.68 (-17.54, -13.83)**	**-27.07 (-29.59, -24.55)**	**-39.87 (-43.31, -36.42)**	**-51.65 (-57.64, -45.67)**
moderate	**-3.68 (-5.50, -1.85)**	**-8.53 (-10.33, -6.72)**	**-15.42 (-18.09, -12.75)**	**-24.42 (-27.82, -21.02)**	**-29.09 (-35.72, -22.45)**

Data are expressed as regression estimate (95% Confidence Interval) of quantile regression model by adjustment for gender, educational level, residence area, geographic location, smoking status, alcohol intake, vegetable consumption and total daily energy intake. Results in bold mean significant at $p<0.05$.
